# Nerves: Their Uses and Troubles

**Published:** 1887-07-16

**Authors:** 


### THE DOCTOR'S PULPIT.

III.—Nerves: their Uses and Troubles.

*(Concluded from p. 253, vol. ii.)*

Some of our less patient readers will think it high time we were coming to the end of our story of the nerves. We are anxious not to weary anybody, because it is with mental food as with material: what is digested does good, not what is eaten; and generally that only is digested which is eaten or read with relish. But there are at least two or three other sets of nerves besides those of the skin, the tongue, the eye, the ear, and the muscles, which ought to be mentioned to make this cursory sketch possess the least approach to completeness.

In this age of indigestion, when fortunes are made by a stomach pill, we should utterly fail of our duty if we said nothing of the nerves of the stomach. Surely, some will say, it is enough to have a stomach, and to know it, without having nerves in it as well. It is because you have nerves in the stomach that you do know it: and these nerves are the greatest friends some men and women have, particularly Aldermen, and women who drink strong tea. The nerves of the stomach are physiological prophets and sword-bearer's, denouncing and inflicting all manner of punishments upon foolish and self-indulgent people. Obey them, and they will make you happy in due time. Disobey them, and they will as certainly make you miserable: and serve you right.

The heart too has its nerves. It is they that make the young lady's heart go pit-a-pat when she hears her lover's rat-at-at-tat on the knocker; and it is they that give Mrs. Robinson such attacks of palpitation when she has swallowed four or five half-pints of strong tea a day for two or three years. We must, however, draw a line somewhere, and we will draw it so as to include the great centres of the nervous system, the brain and spinal cord. The cord is like a telegraph wire. Sensations rush upwards along it from the body to the brain: motions rush downwards from the brain to the body. The cord is not, however, merely a conductor: it is a producer, and a storehouse of nerve-energy as well. But the brain is the grand centre of man which distinguishes him from all other animals. We are not here going to enter into a comparison of the brain of man with that of the chimpanzee, or of the dog, or even to set side by side the thinking organs of the bushwoman and the senior wrangler. There is sufficient resemblance between the brain of the chimpanzee and that of the highest kind of man to make us see that physically, as well as sympathetically, "One touch of nature makes the whole world kin." But there is also divergence great enough to show us that man is placed at the head of a series, and so decidedly at the head that no philosopher who values his reputation will venture to hint a date when any other animal will dispute the headship with him.

The brain is a dreadful organ to be the owner of. It not only enables the tongue to taste, the eye to see, the ear to hear, and the whole body to move, but it gives man the means to think, to reason, to know right from wrong, to look backward to the remotest past, to look forward to a never-ending future. It gives him, to a certain extent, the power to control his own destiny, to make himself great and noble, or to make himself mean and base. Strange conjunction of uses!—it gives him also the power to understand what toothache is, and to suffer from a miserable headache after a too heavy supper or a night's debauch.

The brain and its attendant nerves are by no means Stoics: they "have not that repose which stamps the caste of Vere de Vere." If men hurt them they will complain: or if they are neglected and ill-fed, or have not sufficient rest, they will cry out. What is bodily fatigue?—the crying out of the nerves to tell the man that he has worked enough for that day. What is mental weariness?—the prayer of the nerves for rest and quiet sleep. What are sciatica and other forms of neuralgia?—the shouting and howling of the nerves to warn the owner of them that he is on the very edge of a dangerous break-down in health, and that he had better stop immediately, send for the doctor, and get himself repaired without delay.

If men would but live wisely and in accordance with the principles of health for three generations, neuralgias of all kinds would almost entirely die out. But a physiologist talking like this always feels that he is a kind of John the Baptist—"a voice crying in the wilderness" whom nobody listens to. The city man, eager to make his fortune, goes full gallop over the whole racecourse of life, and never minds the doctor. The man of pleasure, as Tom Hood says, "Puts his thumb unto his nose, and spreads his fingers out" when the physiologist tries to teach him anything. Miss Skin-and-Bone, aged forty-five, draws her waist all the tighter, because the apostle of health ventures to make known his evangel to her: and people generally are so indifferent to the demands of bodily well-being when self-indulgence claims them for its own, that the doctor who is the least akin to the prophet Jeremiah, or to Thomas Carlyle, is tempted to exclaim in despair, "There are four millions of people in London, and nearly forty millions in the country,—mostly fools." However, no man can do more than his best: and no man should, under any circumstances, do less than his best: and acting on these principles, we have ventured to give this brief chapter out of the Gospel of Physiology.

In the treatment of neuralgias and other nerve-affections there is great latitude. For mental and other nerve-worries and excitements the various bromides are used. For nerve-pains the outward application of strong liniments, such as those of aconite, belladonna, and chloroform, is of service. Internally, large doses of quinine—say from seven or eight up to twenty grains, or more—may be given in severe cases every three or four hours. The patient who takes twenty grains will probably think his head is going to burst: but it will not burst if the doctor has selected it properly. (All the methods here recommended are recommended to the doctor, not to the patient.) Quinine and iron, with strychnia and hydrobromic acid, form a good mixture. The chloride of ammonium with iron is an excellent remedy in full doses. Tonga is occasionally good. In the case of severe-sciatica the nerve is sometimes dissected out and stretched. This is barbarous treatment, and seldom gives any great relief. The best treatment of all is to preserve the general vigour of the body: and the next best to take such steps, under the guidance of an intelligent doctor, as will restore that vigour when it is impaired. Speaking broadly, the whole nervous system must be robust and healthy, if every part of it is to be kept free from pain and in a condition to do its duty.

